# Irritable bowel syndrome patients who are not likely to respond to fecal microbiota transplantation

**DOI:** 10.1111/nmo.14353

**Published:** 2022-03-18

**Authors:** Magdy El‐Salhy, Tarek Mazzawi, Trygve Hausken, Jan Gunnar Hatlebakk

**Affiliations:** ^1^ Department of Medicine Stord Hospital Stord Norway; ^2^ Department of Clinical Medicine University of Bergen Bergen Norway; ^3^ Department of Medicine Faculty of Medicine Al‐Balqa Applied University Salt Jordan

**Keywords:** *Alistipes*, fatigue, IBS subtypes, nonresponders, responders, sex

## Abstract

**Background:**

Fecal microbiota transplantation (FMT) interventions have recently been advocated to not succeed in every irritable bowel syndrome (IBS) patient, since the outcome of FMT varies with the IBS subset. This study investigated the factors potentially affecting FMT response using the same patient cohort used in our previous study.

**Methods:**

This study included 109 patients who received allogenic FMT. Patients completed five questionnaires that assessed their symptoms and quality of life at baseline and at 2 weeks, 1 month, and 3 months after FMT. Patients also provided fecal samples at baseline and 1 month after FMT. The fecal bacterial profile and dysbiosis index (DI) were determined using 16S rRNA gene PCR DNA amplification covering variable genes V3–V9. Response to FMT was defined as a decrease of ≥50 points in the total IBS‐SSS score after FMT.

**Results:**

An IBS patient's response or nonresponse to FMT was not determined by age, IBS duration, IBS subtype, IBS symptoms, fatigue, quality of life, or DI. There were more male nonresponders than responders, and the fluorescence signals of *Alistipes* were lower in nonresponders than in responders.

**Conclusions:**

We concluded that IBS patients who are male and/or have low fecal *Alistipes* levels are most likely to not respond to FMT treatment. Whether low fecal *Alistipes* levels could be used as a marker for predicting the outcome of FMT remains to be determined.

www.clinicaltrials.gov (NCT03822299).

## Graphical abstract

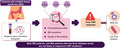

**Key points**

- It has been suggested that the outcomes of FMT in IBS patients vary depending on the IBS subset.
- The present study showed that females are more likely to respond to FMT than males. Age, IBS duration, IBS subtype, IBS symptoms, fatigue, quality of life, or DI did not differ between responders and nonresponders.
- Nonresponders to FMT had lower fecal levels of *Alistipes* than responders at the baseline. Further studies are needed to establish whether fecal levels of *Alistipes* could be used as a marker to identify the IBS subset that is likely to respond to FMT.

## 1 INTRODUCTION

In addition to bacteria, the gut microbiota includes fungi, archaea, viruses, and protozoans.^1^, ^2^ Although bacteria have been detected in the esophagus and stomach, most gut bacteria are located in the intestine.^1^, ^2^ The intestine contains more than 2000 bacteria species that mostly belong to the phyla Firmicutes and Bacteroides.^1^, ^2^, ^3^, ^4^ Gut microbiota are important for health, and disturbing their composition is associated with several gastrointestinal and nongastrointestinal disorders/diseases.^1^, ^2^

The intestinal bacteria of irritable bowel syndrome (IBS) patients deviate from those of healthy subjects, with these patients also having a lower bacterial diversity (dysbiosis).^5^, ^6^, ^7^, ^8^, ^9^, ^10^ This deviation is believed to be involved in the pathophysiology of this disorder.^11^ Fecal microbiota transplantation (FMT) has been performed on IBS patients in seven randomized controlled trials with varying outcomes.^12^ The successful outcome of FMT seems to depend on the donor and the FMT protocol.^12^

FMT intervention has recently been suggested to not succeed in every IBS patient, with the outcomes varying with the IBS subset.^13^ Another IBS intervention, low‐FODMAP (fermentable oligo‐, di‐, mono‐saccharides, and polyols)‐diet, which reportedly improves the symptoms and quality of life in about 50%–75% of IBS patients,^14^ identified a subset that is likely to respond to this intervention.^15^ The subtyping selection was based on microbiota profiles.

This study investigated which factors could affect the FMT responses in the same IBS patient cohort that was used in our previous randomized, double‐blind, placebo‐controlled study.^16^

## 2 MATERIALS AND METHODS

### 2.1 Study design

The design of this study has previously been described in detail.^16^ To summarize, patients completed five questionnaires that assessed their symptoms and quality of life at baseline and at 2 weeks, 1 month, and 3 months after FMT. Patients also provided fecal samples at baseline and 1 month after FMT. The patients were randomized at a 1:1:1 ratio into placebo (own feces), 30‐g (donor feces), and 60‐g (donor feces) groups.^16^ Fecal samples were immediately frozen and stored at –80°C. The FMT process has previously been described in detail.^16^ To summarize, the transplant was mixed manually with sterile saline and was administered to the distal duodenum via a gastroscope.^16^

### 2.2 Patients and donor

This study included the 109 patients who received allogenic FMT (30 g and 60 g of donor feces) according to our previous study.^16^ Table 1 lists the characteristics of these patients, who are described in detail elsewhere.^16^ The inclusion criteria were an age between 18 and 75 years and having moderate‐to‐severe IBS symptoms, as indicated by an IBS Severity Scoring System (IBS‐SSS) score of ≥175. The exclusion criteria were those who were pregnant or planning pregnancy, lactating, had a systemic disease, had immune deficiency, were receiving treatment via immune‐modulating medication, had a psychiatric illness, excessively consumed alcohol, or abused drugs. Patients who took probiotics, antibiotics, or IBS medications within 8 weeks prior to the study were also excluded.^16^

**TABLE 1:** Characteristics of irritable bowel syndrome (IBS) patient who respond and do not respond to fecal microbiota transplantation

	Overall	Responders	Nonresponders
Number	109	90	19
Age, years (mean ± SD)	38.6 ± 12.5	38.5 ± 12.6	39.1 ± 12.3
Sex (female/male)	133/32	75/15	10/9
IBS‐D	41	35	6
IBS‐C	40	32	8
IBS‐M	28	23	5
IBS duration, years (mean ± SD)	18.9 ± 9.7	19.5 ± 10.0	16.2 ± 8.1

Note

IBS‐D, diarrhea‐predominant IBS; IBS‐C, constipation‐predominant IBS; IBS‐M, mixed‐diarrhea‐and‐constipation IBS.

The donor used in this study has also been previously described in detail.^16^ To summarize, he was screened according to the European guidelines for FMT donors.^7^, ^17^

He was a healthy male aged 36 years who fulfilled the clinical criteria of a superdonor.^12^, ^16^

### 2.3 Symptom and quality‐of‐life assessments

This study used the IBS‐SSS, Birmingham IBS Symptom, Fatigue Assessment Scale (FAS), IBS Quality of Life Scale (IBS‐QoL), and the Short‐Form Nepean Dyspepsia Index (SF‐NDI) questionnaires.^18^, ^19^, ^20^, ^21^, ^22^, ^23^, ^24^ FMT response was defined as a decrease of ≥50 points in the total IBS‐SSS score after FMT.

### 2.4 Microbiome analysis and dysbiosis index

The fecal bacteria profile and dysbiosis index (DI) were determined using 16S rRNA gene PCR DNA amplification covering variable genes V3–V9. Probe labeling was achieved by single‐nucleotide extension and signal detection using the BioCode 1000A 128‐Plex analyzer (Applied BioCode).^8^ The 48 employed bacterial markers detected bacteria within 5 phyla (Firmicutes, Proteobacteria, Bacteroidetes, Tenericutes, and Verrucomicrobia) and assessed >300 bacteria at different taxonomic levels.^8^, ^9^ DI was measured on a 5‐point scale from 1 to 5, where DIs of 1 and 2 were normobiosis, and 3–5 were dysbiosis.^8^

### 2.5 Statistical analysis

Differences between females and males and with IBS subtypes were analyzed using the *χ* ^2^ and Fisher's exact tests. Differences in the total scores on the IBS‐SSS, Birmingham IBS Symptom, FAS, IBS‐QoL, SF‐NDI, DI, and in fluorescence signals of fecal bacteria between responders and nonresponders were analyzed using the Mann–Whitney test. These analyses were performed using GraphPad Prism (version 8.0).

### 2.6 Ethics

The study was approved by the Western Regional Committee for Ethics, Bergen, Norway (approval no. 2017/1197/REK vest). All subjects provided both oral and written consents prior to participation. The study was registered at www.clinicaltrials.gov (NCT03822299).

## 3 RESULTS

### 3.1 Response to FMT

**FIGURE 1:**
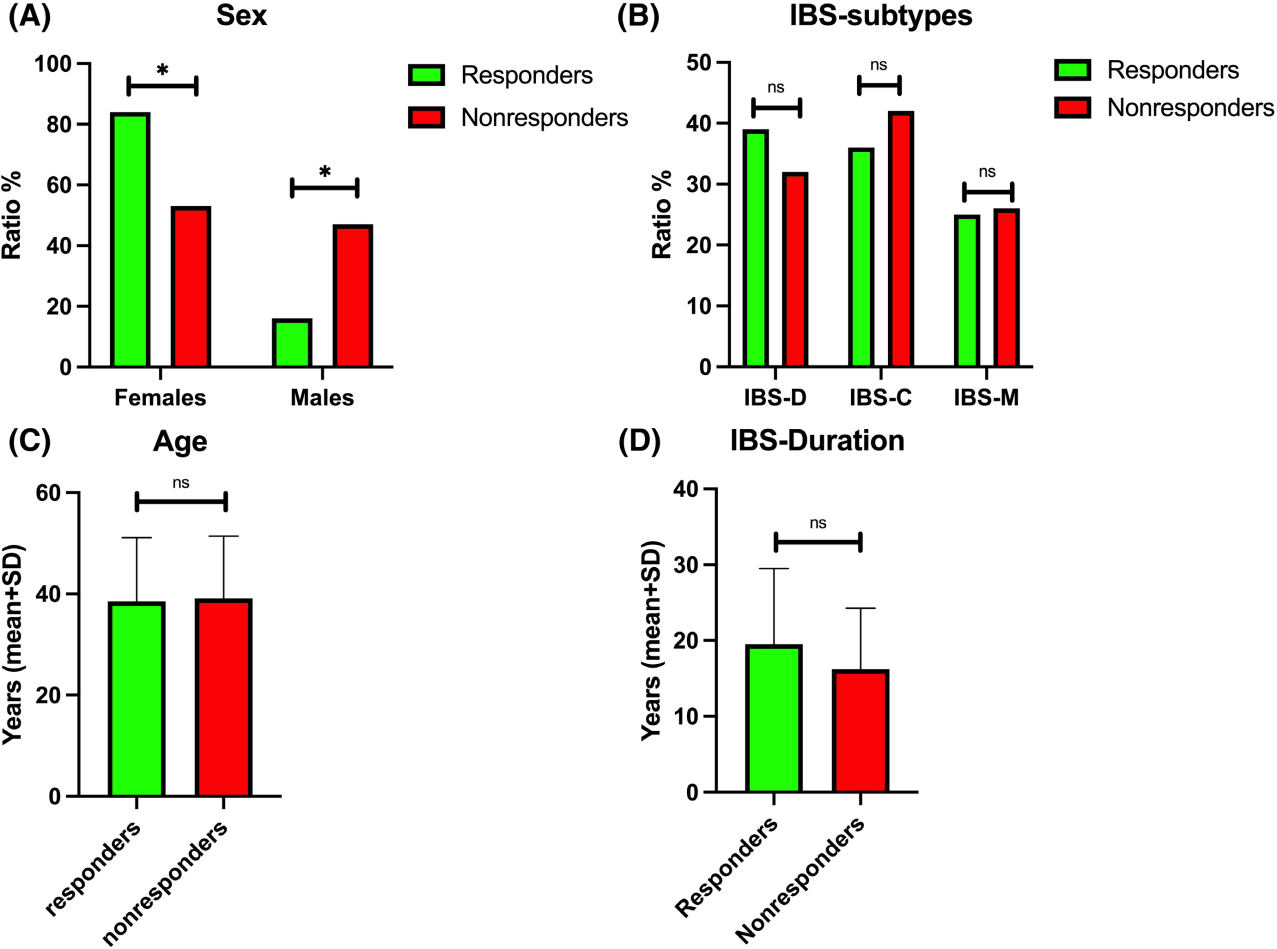
Differences between responders and nonresponders in sex (A), proportions of IBS subtypes (B), age (C), and irritable bowel syndrome (IBS) duration (D). ns, not significant; ^*^, *p* < 0.05

The response rates in males were significantly lower than in females (*p *= 0.006). Whereas, in responders, the proportion of females was significantly higher than males, in nonresponders the proportion of males was significantly higher than females (Figure 1). There were no differences between responders and nonresponders in IBS subtypes, age, or IBS duration (Figure 1).

### 3.2 Symptoms and quality of life

**FIGURE 2:**
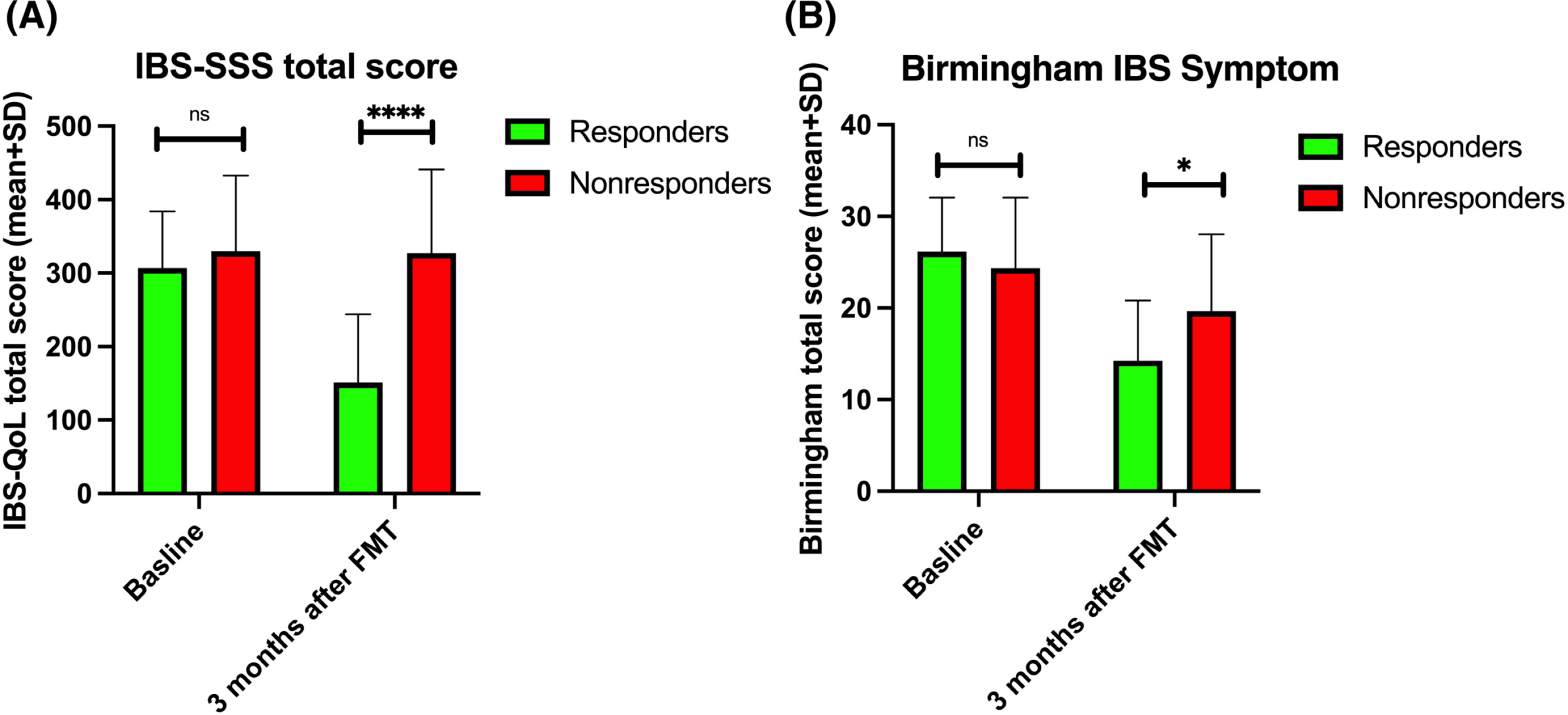
Total IBS Severity Scoring System “IBS‐SSS” (A), and Birmingham IBS Symptom (B) scores in responders and nonresponders at baseline and at 3 months after fecal microbiota transplantation (FMT). ns, not significant; ^*^ *p* < 0.05; ^****^ *p* < 0.0001

**FIGURE 3:**
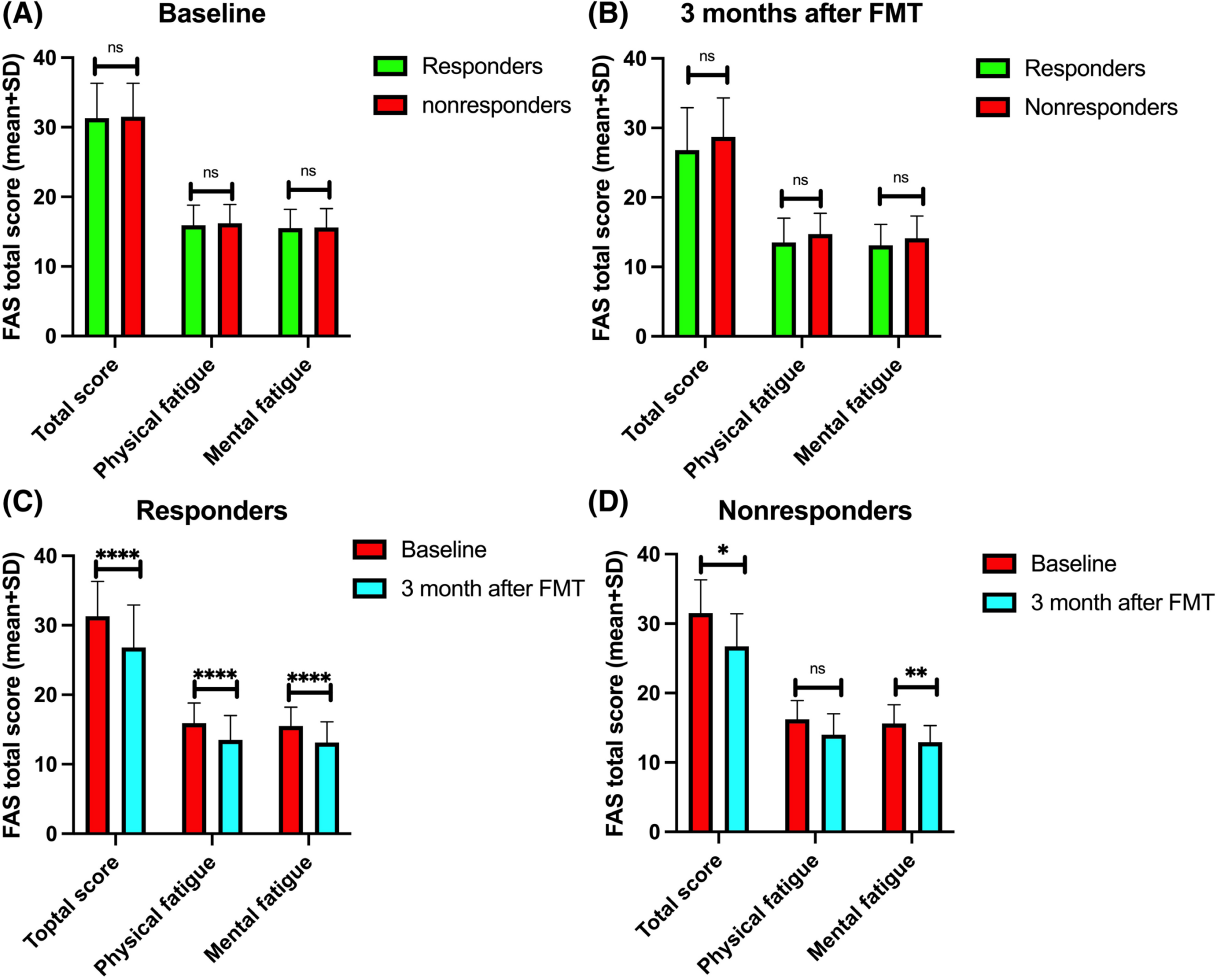
Total Fatigue Assessment Scale (FAS) scores and its two subitems in responders and nonresponders at baseline (A) and at 3 months after FMT (B). Changes in total FAS scores and scores for its subitems in responders (C) and nonresponders (D) at the baseline and 3 months after FMT. ns, not significant; ^*^ *p* < 0.05; ^**^ *p* < 0.01; ^****^ *p* < 0.0001

The total IBS‐SSS and Birmingham IBS Symptom scores did not differ between responders and nonresponders at baseline, but decreased among responders 3 months after FMT (Figure 2). The total FAS scores and the scores for its two subitems did not differ between responders and nonresponders at baseline and at 3 months after FMT (Figure 3). Total FAS scores significantly decreased in both responders and nonresponders at 3 months after FMT. Meanwhile, the physical and mental fatigue of responders decreased, while only the mental fatigue of nonresponders decreased (Figure 3).

**FIGURE 4:**
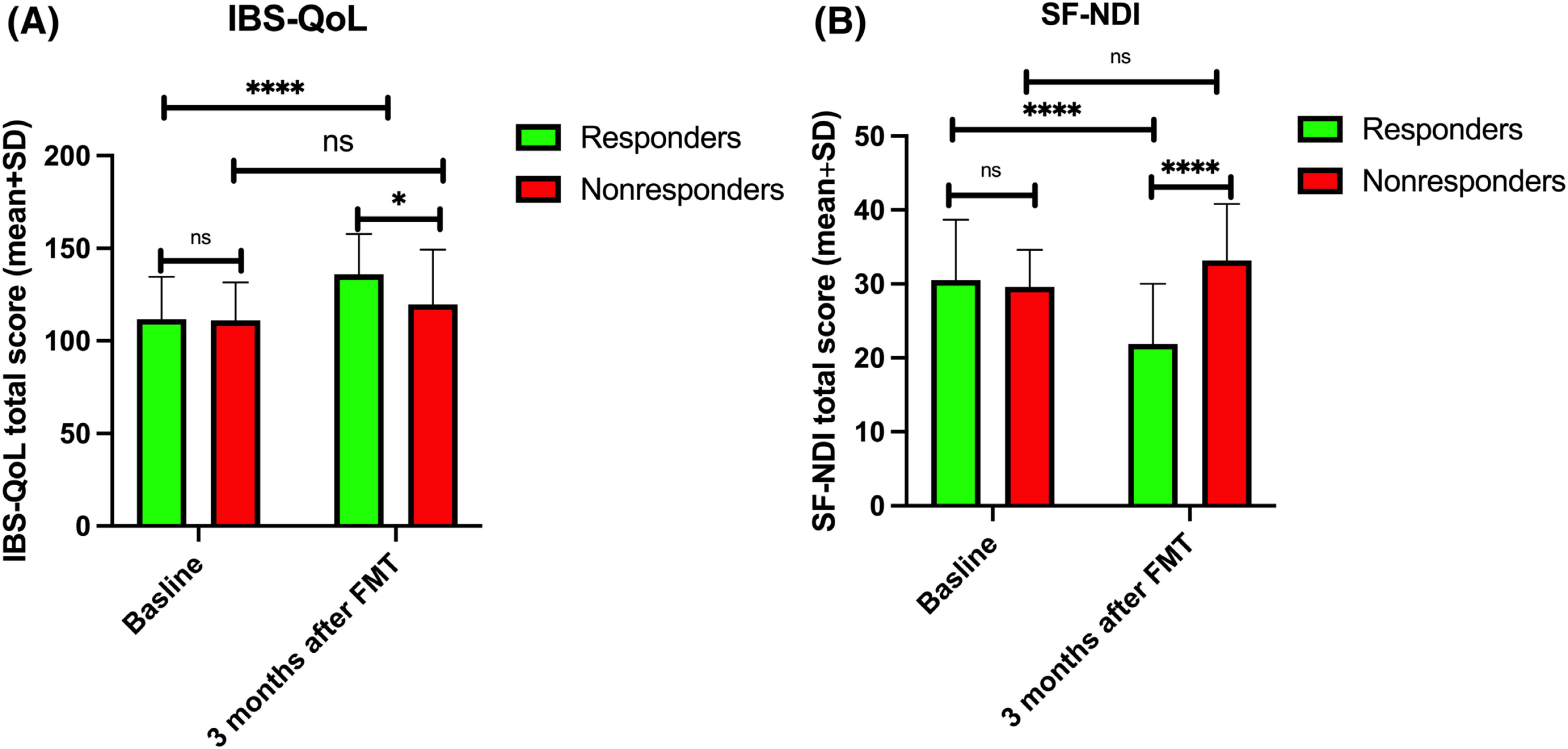
Total Quality of Life Scale (A) and Short‐Form Nepean Dyspepsia Index (B) scores of responders and nonresponders at baseline and at 3 months after FMT. ns, not significant; ^*^ *p* < 0.05; ^****^ *p* < 0.0001

The total IBS‐QoL and SF‐NDI scores did not differ between responders and nonresponders at baseline (Figure 4). The scores for both IBS‐QoL and SF‐NDI changed in responders but not in nonresponders after FMT (Figure 4).

### 3.3 Bacteria analysis

**FIGURE 5:**
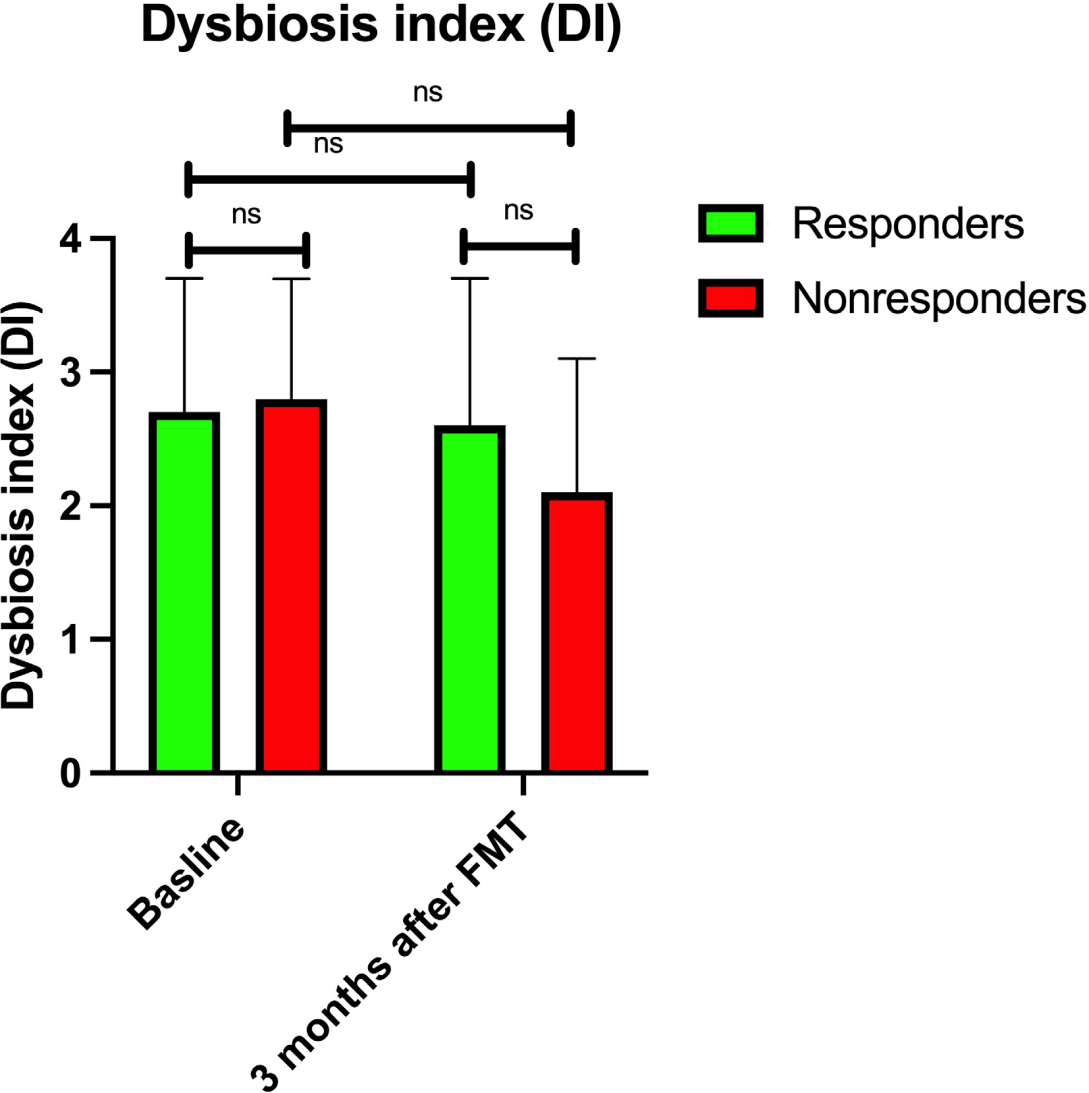
Dysbiosis index (DI) of responders and nonresponders at baseline and at 1 month after FMT. ns, not significant

DIs did not differ between responders and nonresponders at baseline and at 3 months after FMT (Figure 5).

**FIGURE 6:**
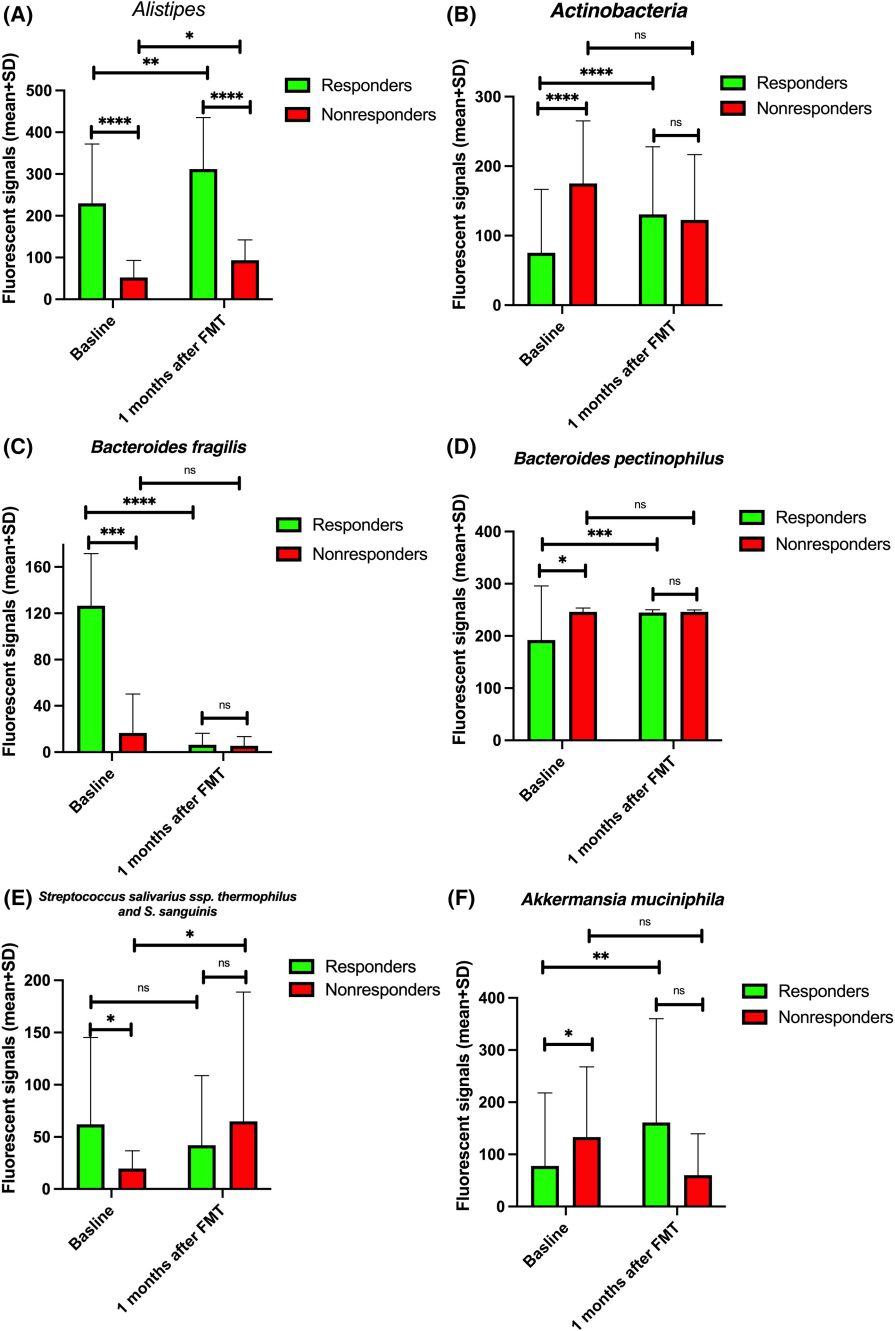
Fluorescence signals of *Alistipes* (A), *Actinobacteria* (B), *Bacteroides fragilis* (C), *Bacteroides pectinophilus* (D), *Streptococcus salivarius* ssp. *thermophilus* and *Streptococcus sanguinis* (E), and *Akkermansia muciniphila* (F) of responders and nonresponders at baseline and at 1 month after FMT. ns, not significant; ^*^ *p* < 0.05; ^**^ *p* < 0.01; ^***^ *p* < 0.001; ^****^ *p* < 0.0001

**FIGURE 7:**
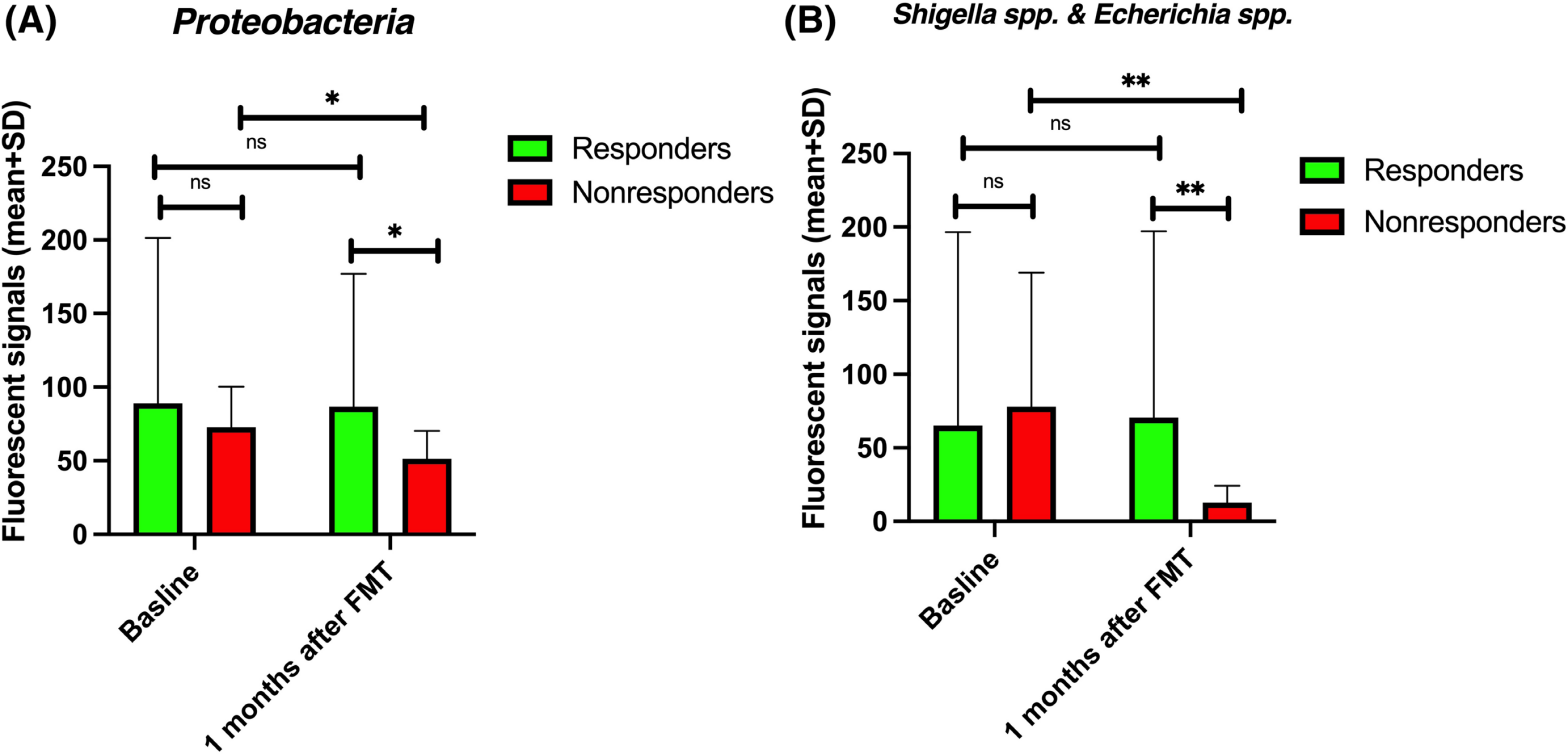
Fluorescence signals of *Proteobacteria* (A) and *Shigella* spp. and *Escherichia* spp. (B) at baseline and at 1 month after FMT. ns, not significant; ^*^ *p* < 0.05; ^**^ *p* < 0.01

At baseline, the fluorescence signals of six bacteria differed between responders and nonresponders (Figure 6). These bacteria were *Alistipes*, *Actinobacteria*, *Bacteroides fragilis*, *Bacteroides pectinophilus*, *Streptococcus salivarius* ssp. *thermophilus*, *and sanguinis*, and *Akkermansia muciniphila*. The fluorescence signals of *Alistipes*, *Bacteroides fragilis*, *Streptococcus salivarius* ssp. *thermophilus*, and *Streptococcus sanguinis* were higher in responders than in nonresponders, and the fluorescence signals of *Actinobacteria*, *Bacteroides pectinophilus*, and *Akkermansia muciniphila* were lower in responders than in nonresponders (Figure 6). There were no differences in the fluorescence signals of all these bacteria except for *Alistipes* after FMT. Although the fluorescence signals of *Alistipes* increased significantly after FMT in nonresponders, it never reached the levels of the responders (Figure 6). The fluorescence signals of *Proteobacteria*, *Shigella* spp., and *Escherichia* spp. decreased in nonresponders at 1 month after FMT (Figure 7).

## 4 DISCUSSION

There is currently no effective treatment for IBS, and so the available clinical treatments focus on symptom relief. Applying FMT with a carefully selected donor and with a proper protocol is a promising intervention.^12^ A single FMT improves abdominal symptoms, fatigue, and increases the quality of life in most treated patients for at least 1 year.^25^ Moreover, when this intervention is done properly, it is safe despite the possibility of mild self‐limiting adverse events.^12^, ^16^, ^25^ Restricting this effective treatment to specific IBS subsets has been discussed.^13^

The present study found no differences between responders and nonresponders regarding age, IBS duration, IBS subtype, IBS symptoms, fatigue, quality of life, or DI. However, most nonresponders were males. Recently published studies have indicated that males respond to FMT less than females.^13^, ^26^ Speculating about the cause of this sex‐related difference in FMT responses is difficult, but it could be due to differences in social, psychological, and/or biological factors between females and males. It should also be remembered that the prevalence of IBS in females is double that in males.^27^

The fluorescence signals of six different bacteria differed between responders and nonresponders at baseline. These bacterial differences disappeared after FMT, except for those of *Alistipes*. The fluorescence signals of *Alistipes* were lower in nonresponders than in responders at baseline, and they increased after FMT in nonresponders, but never reached the levels of the responders. *Alistipes* levels have been reported to be lower at baseline in those who responded to FMT after 3 months but relapsed after 1 year than in those who maintained a response 1 year after FMT.^25^ Furthermore, *Alistipes* levels were negatively correlated with total IBS‐SSS and FAS scores.^25^ *Alistipes* is a genus of bacteria that was described in 2003. It is anaerobic, gram‐negative, rod‐shaped, and does not form spores.^28^ *Alistipes* levels are associated with several diseases such as depression, anxiety, chronic fatigue syndrome, autism, cirrhosis, and aging.^28^ Several immuno‐inflammatory and metabolic mechanisms have been proposed for its role in human health and its association with diseases.^28^ *Alistipes* hydrolyze tryptophan to indole. As tryptophan is a precursor for serotonin, an increase in level of *Alistipes* would decrease serotonin availability.^28^ *Alistipes* has also been shown to express glutamate decarboxylase, an enzyme that metabolizes glutamate into γ‐aminobutyric acid (GABA) in chickens.^28^ Moreover, *Alistipes* produces the short chain fatty acids (SCFAs) acetic, succinic and propionic acids.^28^, ^29^ These SCFAs have anti‐inflammatory effects.^28^ Propionic acid has neurobiological effects in rats.^30^Furthermore, *Alistipes* expresses methylmalonyl‐CoA epimerase, in which the gene for this enzyme is located on an operon with the acetyl‐CoA carboxylase gene.^28^

While no improvements were found for nonresponders regarding abdominal symptoms or quality of life, fatigue did improve, in terms of mental fatigue. The fluorescence signals of *Alistipes* increased after FMT in nonresponders, and *Alistipes* is known to affect mental health.^28^ It is yet to be determined to what extent this change in *Alistipes* levels contributes to this improvement.

In conclusion, this study has revealed that male IBS patients and IBS patients with low fecal *Alistipes* levels are not likely to respond to FMT treatment. Further studies are needed to establish whether *Alistipes* levels can be used as indicator to the FMT outcome.

The strengths of this study are that it included a relatively large IBS patient cohort (delet), comprising three IBS subtypes, males and females, and used a single well‐defined donor. These allowed a sub‐group analysis. Limitations of this study are that it did not include the fourth IBS subtype, IBS‐U, and it only investigated predetermined targets of the intestinal bacterial contents.

## CONFLICT OF INTEREST

The authors have nothing to disclose.

## AUTHOR CONTRIBUTIONS

M.E.S. designed the study, obtained the funding, administered the study, recruited the patients, performed FMT, collected, analyzed, and interpreted the data, and drafted the manuscript. T.M., T.H., and J.G.H. contributed to the design of the study, to data analysis, and critically revised the manuscript for important intellectual content.
